# Hexagonal Prisms Form in Water‐Ice Clouds on Mars, Producing Halo Displays Seen by Perseverance Rover

**DOI:** 10.1029/2022GL099776

**Published:** 2022-09-09

**Authors:** M. T. Lemmon, D. Toledo, V. Apestigue, I. Arruego, M. J. Wolff, P. Patel, S. Guzewich, A. Colaprete, Á. Vicente‐Retortillo, L. Tamppari, F. Montmessin, M. de la Torre Juarez, J. Maki, T. McConnochie, A. Brown, J. F. Bell

**Affiliations:** ^1^ Space Science Institute Boulder CO USA; ^2^ Instituto Nacional de Técnica Aerospacial Madrid Spain; ^3^ Jet Propulsion Laboratory California Institute of Technology Pasadena CA USA; ^4^ Mullard Space Science Laboratory University College London London UK; ^5^ NASA Goddard Space Flight Center Greenbelt MD USA; ^6^ NASA Ames Research Center Moffett Field CA USA; ^7^ Centro de Astrobiología (INTA‐CSIC) Madrid Spain; ^8^ LATMOS Paris France; ^9^ Plancius Research Severna Park MD USA; ^10^ Arizona State University Tempe AZ USA

## Abstract

Observations by several cameras on the Perseverance rover showed a 22° scattering halo around the Sun over several hours during northern midsummer (solar longitude 142°). Such a halo has not previously been seen beyond Earth. The halo occurred during the aphelion cloud belt season and the cloudiest time yet observed from the Perseverance site. The halo required crystalline water‐ice cloud particles in the form of hexagonal columns large enough for refraction to be significant, at least 11 μm in diameter and length. From a possible 40–50 km altitude, and over the 3.3 hr duration of the halo, particles could have fallen 3–12 km, causing downward transport of water and dust. Halo‐forming clouds are likely rare due to the high supersaturation of water that is required but may be more common in northern subtropical regions during northern midsummer.

## Key Points

- A 22°‐radius halo around the Sun was imaged for over 3 hr by cameras on the Perseverance rover on Mars
- Such a halo is diagnostic of scattering by large, hexagonal water‐ice crystals and has not previously been seen beyond Earth
- The halo implies that high supersaturation of water may be more common in late summer in the northern subtropics than elsewhere

## 1 Introduction

Water‐ice clouds are common in Mars's northern tropics around aphelion, comprising 3–4 µm radius particles (Clancy et al., 2003) that have not been shown to have the diversity of shapes and optical effects seen on Earth. Martian cloud particles have been modeled as complex polyhedra that resemble precursors of terrestrial cirrus cloud particles (Wolff et al., 2019). Terrestrial ice clouds typically develop further into an assortment of hexagonal columns and plates, along with aggregates and more complex shapes (Bailey & Hallet, 2008). Terrestrial clouds can produce a wide variety of halos, including those caused by refraction through faces inclined 60° and 90° to one another in crystals that can be randomly or preferentially oriented (Minnaert, 1974). The most common terrestrial halo is at 22° from the Sun and is caused by randomly oriented hexagonal columns (prisms) and rosettes (clusters of hexagonal prisms) in which the refraction is through faces inclined at 60° (Barran, 2009). Due to the near‐Sun geometry, this halo is inaccessible to orbiting spacecraft; no halos have been reported in images by landers on other worlds.

There has been little evidence that Martian cloud particles make the transition to hexagonal prisms or attain sizes such that refraction could produce a halo. Whiteway et al. (2009) found fall streaks seen by the Phoenix LIDAR to be consistent with 42 × 127 μm water‐ice columns, but no halos were reported in Phoenix images despite frequent clouds. This may mean that the large particles were not common, or they were irregular and did not include the necessary prism. Cooper et al. (2019, 2020) used Curiosity rover images and Mars Color Imager (MARCI) data to suggest the presence of columns. In the case of the Curiosity images, the determination was based on the statistics of radiance differences as clouds moved in the sky—there was no halo imaged—and included 20°–50° scattering angles. While no halo feature was detected in the retrieved phase function, the columnar shape was argued based on the absence of features corresponding to other shapes at larger angles. In the case of the MARCI data, the orbital geometry limited the observations to non‐halo angles and the determination was also based on the absence of features from the alternative particles considered. While Cooper et al. (2020) considered aphelion cloud belt (ACB) particles to be 2.75 μm radius, no size constraint was presented.

The Perseverance rover landed in Jezero crater at 77.5°E longitude, 18.4°N latitude on 18 February 2021 and began environmental surveys among other activities. In this work, we show that a water‐ice halo was unambiguously visible on sol 292 (a sol is a Martian solar day) at solar longitude (*L*_S_) 142° of Mars year 36 (see Clancy et al., 2000, for Mars time conventions). The halo, also potentially visible on a few additional sols, requires the existence of previously unexpected large, crystalline particles. In Section 2, we describe imaging campaigns undertaken with Perseverance cameras and the methods by which we analyzed the data. In Section 3, we report on images of a halo by three instruments, comprising four separate cameras, and discuss the implications. Section 4 summarizes conclusions.

## 2 Data and Methods

### 2.1 Imaging Campaigns

Several instruments on the Perseverance rover have been used for routine reconnaissance of the environment. Cameras involved in the halo detection are Navcam, Mastcam‐Z, and Skycam. The first two reside on the rover's remote sensing mast, which aims them, while the last resides on the rover's deck and is always aimed upward.

Navcam is an engineering camera designed to support rover navigation (Maki et al., 2020). It has a 96° × 72° field of view (FOV) with a 5,120 × 3,840 pixel array using a Bayer pattern of microfilters for red, green, and blue (RGB) color images. The images used in this study were 1,280 × 960 RGB images spanning the full FOV. The Navcam cloud survey used five images to acquire a nearly full sky mosaic.

Mastcam‐Z is a multispectral and stereo camera designed for geologic and atmospheric science (Bell et al., 2021). Each camera, left and right, has a variable FOV depending on the zoom setting. The sensor has a 1,600 × 1,200 array of pixels using a Bayer pattern of microfilters for color when using an infrared‐blocking filter. For monitoring atmospheric optical depth, solar images were acquired using neutral density (ND) filters together with an IR‐blocking filter for RGB images or a narrow 880 nm filter. Sky images were acquired with some optical depth measurements.

Skycam is part of the Mars Environmental Dynamics Analyzer (MEDA, Rodriguez‐Manfredi et al., 2021). It has a 1,024 × 1,024 pixel array containing a 126°‐diameter FOV using fisheye optics and a circular baffle. It acquires monochrome images with an effective wavelength of 691 nm. An ND annulus on the camera window allows sky imaging while also allowing unsaturated solar imaging in mid‐morning and mid‐afternoon. Skycam images taken while the Sun was within the ND annulus were used to track diurnal variation of optical depth and look for transient features in the sky.

### 2.2 Image Processing

Perseverance image headers contain geometric information sufficient to determine the pointing of each pixel, subject to the rover's attitude uncertainty. That uncertainty was near 0.5°, based on the scatter in position of the Sun in Mastcam‐Z images aimed at the Sun. Mosaics of images calibrated to radiance and ratio images were used to look for time‐variable features. In addition, time‐variable features were identified after dividing out a smooth sky radiance profile determined by a regression of the natural logarithm of radiance that was second order in scattering angle and cosine of the zenith angle.

### 2.3 Optical Depth Processing

The use of solar images to determine visible optical depth for tracking dust and ice followed the template described in Lemmon et al. (2015, 2019): the images were calibrated to radiance on sensor; a synthetic aperture was defined around the Sun for photometric measurements of flux; the flux was converted to normal optical depth using the estimated top‐of‐atmosphere flux for each sensor along with the specific image geometry.

For Mastcam‐Z, Hayes et al. (2021) described the instrument calibration. For optical depth processing, the left‐eye RGB images were separated to the three colors (480, 544, and 630 nm), and the right‐eye images added 880 nm. As with previous cameras, the ability to image at different times of day was used for a Beer‐Lambert law fit, which determined what each band would have been for a hypothetical no‐optical‐depth case. The fit was done using mean radiance of the solar disk, to account for the varying Mars‐Sun distance. No temperature corrections were needed, as these were included in the calibration, and no variability of dust on the optics was observed.

For Skycam, Rodriguez‐Manfredi et al. (2021) describe the instrument calibration. The 691 nm optical depth processing could not use the Beer‐Lambert law relative calibration due to the limited geometry of Sun images. Rather, all close occurrences of Skycam and Mastcam‐Z optical depth measurements were used to determine the top‐of‐atmosphere flux required for Skycam to reproduce the Mastcam‐Z measurements. That value has not showed a significant trend during the mission.

## 3 Results

### 3.1 Sol 292 Halos

**Figure 1:**
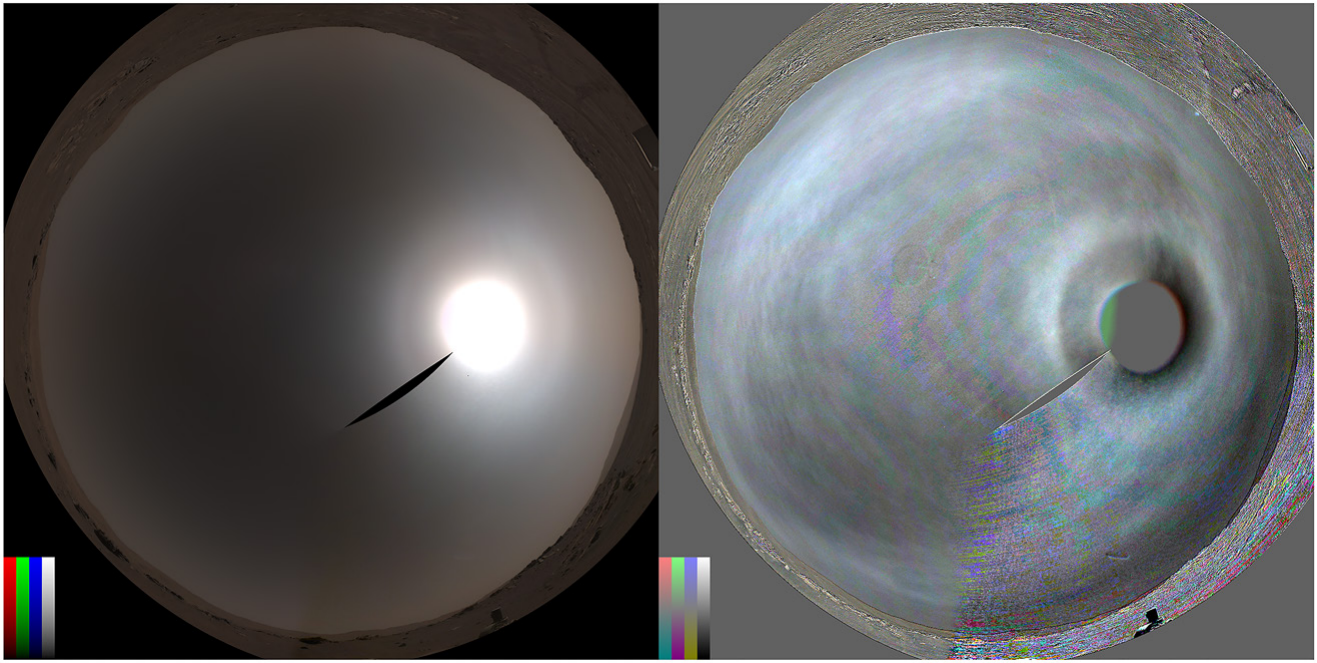
The sol 292 Navcam sky panorama is shown in equidistant projection (north at top, east to the right, zenith centered) on the left, and the ratio of sol 292–299 is shown on the right. Color‐bars show radiance of 0–0.3 (left) and contrast of −20% to 20% (right).

A Navcam cloud survey at 08:57–09:01 local true solar time (LTST) on sol 292 (15 December 2021) showed an apparent ring around the Sun in the 3 out of 5 images that were aimed near the Sun (Figure 1). We identified the ring, which was visible in unprocessed raw images, as a candidate water‐ice halo based on its scattering angle of approximately 22°. We initiated a campaign to attempt to repeat the halo image or identify it as an artifact; observations in support of this were undertaken on sols 299, 303, 304, and 308. No halo was identified in the follow‐up imaging in either Navcam or the supporting Mastcam‐Z images.

The follow‐up imaging was nonetheless useful in that several Navcam surveys were taken with nearly identical geometry to the sol 292 survey and no halo‐like artifacts were seen. (No preceding survey was taken with similar geometry, given the dependence on both time of sol and the rover's orientation). Figure 1 shows the ratio of the sol‐292 survey to the sol‐299 survey. The halo stands out clearly, although other artifacts are present (e.g., noise from underexposure in lower right). While the camera has shown arcuate and streak‐like artifacts depending on the specific position of the Sun: (a) none have resembled the sol 292 images; (b) three images with different geometries showed a halo on sol 292; and (c) several surveys that repeated the sol‐292 geometries did not show a halo or similar artifacts (see also Figures S1 and S2 in Supporting Information S1). Thus, we conclude that the feature observed on sol 292 was a real scattering feature in the Martian sky.

**Figure 2:**
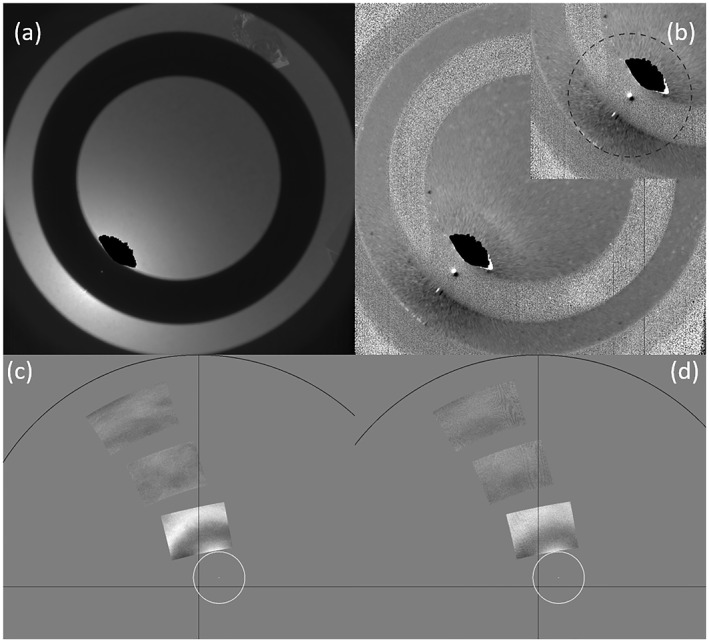
Skycam image for morning of sol 292 (a) with ratio to sol 299 (b). The inset in (b) shows the near‐Sun quadrant with a 22° radius circle around the Sun. The halo fragment is above the Sun. Sol 292 Mastcam‐Z images using the L6 (442 nm) filter (c) and the R2 (866 nm) filter (d) are shown in equidistant projection after dividing out a model background, with the horizon and N‐S and E‐W lines shown in black. The Sun is centered in the 10° white circles.

In a review of other available data from sol 292, we determined that halos could be seen in both Mastcam‐Z and Skycam images (Figure 2). The only Mastcam‐Z morning sky images between sols 290 and 299 were on sol 292 at 11:26 LTST. Figure 2 shows the 440 and 860 nm images in equidistant (fisheye) projection as ratio images that have a low‐frequency polynomial fit divided out. A bright feature with inner edge near 22° is evident in each of the Mastcam‐Z left and right cameras.

Skycam images were acquired regularly in the mornings at 08:34 LTST. While no halo is visible in the unprocessed image, the ratio with the sol‐299 image shows an arcuate feature above the Sun (Figure 2). We do not find the image (ratio) by itself to be sufficient evidence to demonstrate there are halos on Mars. However, given the presence of a halo that morning, we do find the image convincing that the halo was likely present as early as 08:34 LTST. A Skycam image at 14:33 LTST was the only image that sol with appropriate geometry that did not show a halo.

The MEDA Radiation and Dust Sensor (RDS) includes not just Skycam but also a series of photodiodes that take data at 1‐Hz while MEDA is operating (Apestigue et al., 2022; Rodriguez‐Manfredi et al., 2021). The RDS Lateral‐4 sensor was looking 20° (with a 5° FOV) above the rover deck and 24° from the Sun at 08:23 LTST and showed a peak in brightness, relative to other sols (see also Figures S3 and S4 in Supporting Information S1).

### 3.2 Other Candidate Halos

We found no compelling halo candidates in Navcam images other than on sol 292. Mastcam‐Z sky images on sols 257 (12:51 LTST) and 258 (12:06 LTST) may show more subtle halos. Halos were not found above the few percent detection level in most other Mastcam‐Z sky images at 22° scattering angle. Some Mastcam‐Z images have reflection artifacts that may or may not be masking a halo. Skycam images around 08:30 LTST on sols 293–298 show weaker halo‐like features than on sol 292 (Figure S2 in Supporting Information S1).

In addition to the 22°‐halo, water‐ice particles have a halo at 46° and carbon‐dioxide‐ice particles have halos at 26° and 39° (Cowley & Schroeder, 1999). None of these halos were identified in the images with the 22° halo or other images in this period. Both cloud compositions can produce other optical effects, none of which were observed.

### 3.3 Context

Morning ice hazes of optical depth ∼0.1 were common through the Perseverance mission through sol 275, which represents solar longitudes (*L*_S_) of 6°–134°, or northern spring and early summer. Much of this period overlapped with activity of the ACB (Tamppari et al., 2000; Wolff et al., 2019). Like at other landing sites (Lemmon et al., 2015, 2019), optical depth had declined toward a typical mid‐summer (post‐aphelion) minimum.

**Figure 3:**
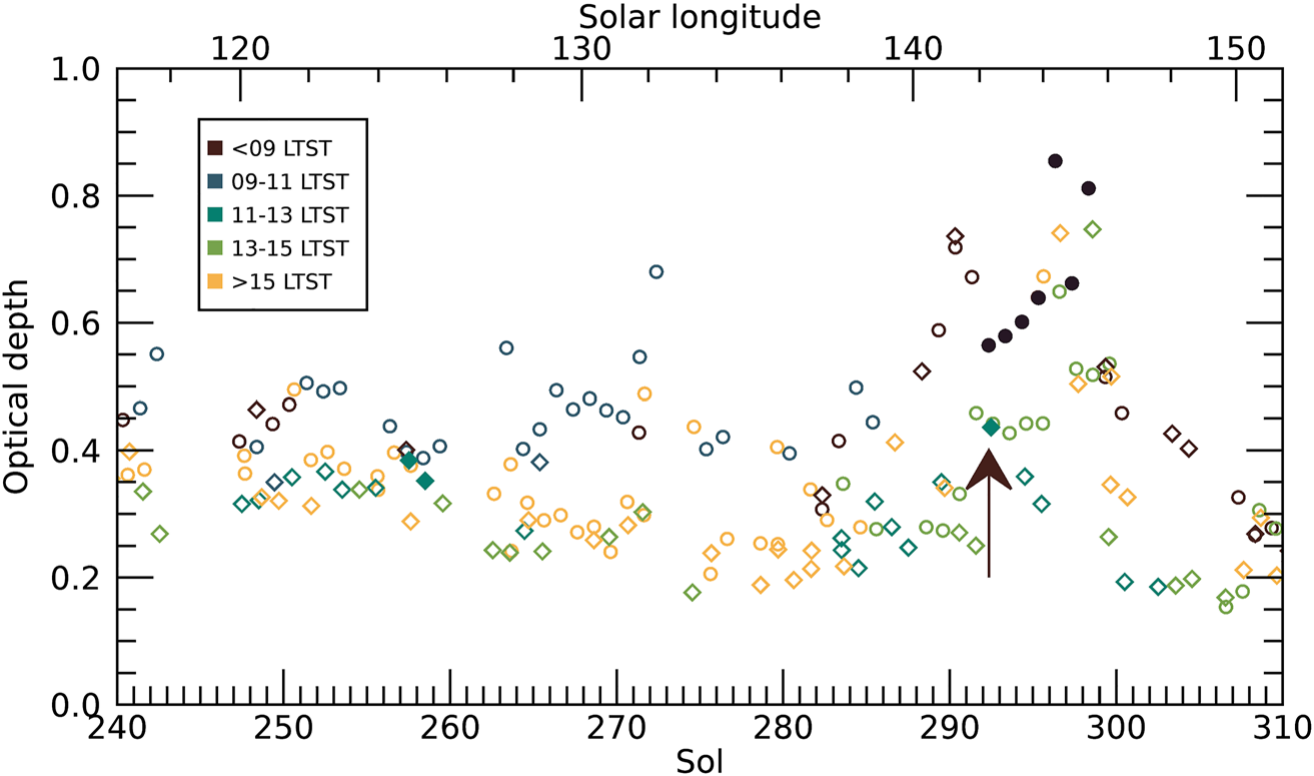
Column optical depth is shown for Skycam (circles) and Mastcam‐Z green channel (diamonds). Filled symbols indicate possible halos associated with optical depth measurements. Colors indicate local true solar time (LTST) according to the legend. Arrow indicates Navcam halo detection.

Around sols 280–285 (*L*_S_ 136°–139°), column optical depth started to become larger and variable at all times of sol (Figure 3). The rising trend continued through the mid‐290s (sol 292 was *L*_S_ 142°), while occasional low values near 0.3 remained. Midsol and afternoon optical depth returned to low values around sol 300 (*L*_S_ 147°), with morning values following by sol 305. Clouds were commonly observed in sky images and near‐horizon parts of landscape images over sols 280–300 (see Figure S5 in Supporting Information S1).

The altitude of the halo‐producing cloud is not determined but may be near 40 km. Pre‐dawn RDS zenith photometry on sol 292 showed unusual sky brightness with the Sun below the horizon (see also Figures S6 and S7 in Supporting Information S1). A 950 nm brightness inflection at ∼94° solar zenith angles suggested the possible presence of a cloud layer at altitudes of ∼44 km, although the data do not rule out additional clouds at lower altitudes. At Gale crater, high‐altitude, morning clouds have been estimated to move from 30 to 45 km altitude to >40 km altitude over *L*_S_ 120°–150°, while geometric techniques were used to measure cloud altitudes from 16.9 to 54.9 km over *L*_S_ 135°–142° (Campbell et al., 2020).

The halo‐forming region was likely of order hundreds of km in extent. At any moment, the lateral extent of the ice‐cloud must have been 2–3 times the altitude of the clouds to form a complete halo. The 3.3 hr duration of the halo implies an extent of one to a few hundred km for realistic wind speeds.

### 3.4 Halo Properties

Halos are geometric‐optics (refraction) features that do not exist for cloud particles small compared to wavelength and thus the presence of halos sets a lower limit for particle size. Bi and Yang (2014) showed T‐Matrix calculations of the transition for compact prisms with length (*L*) of twice the size of a hexagon facet (so, *L* ∼ *D*, diameter). They found that the halos emerged and then became insensitive to size over the size parameter (2π*L*/λ, where λ is wavelength) range of 80–150. For green light, that is *L* of 7–13 μm; at 866 nm, that is 11–21 μm. Thus, we estimate 11 μm to be the minimum length and diameter. The unseen 46° halo is more prominent for plates (*L* < *D*) and less prominent for columns (*L* > *D*), thus we find columns more likely. Aggregates of columns and rosettes comprising large columns also show halos. For arbitrarily large particles, surface roughness may degrade or eliminate the halos (Yang et al., 2008). Thus, we do not consider the halo to be diagnostic of size above the minimum allowed value.

**Figure 4:**
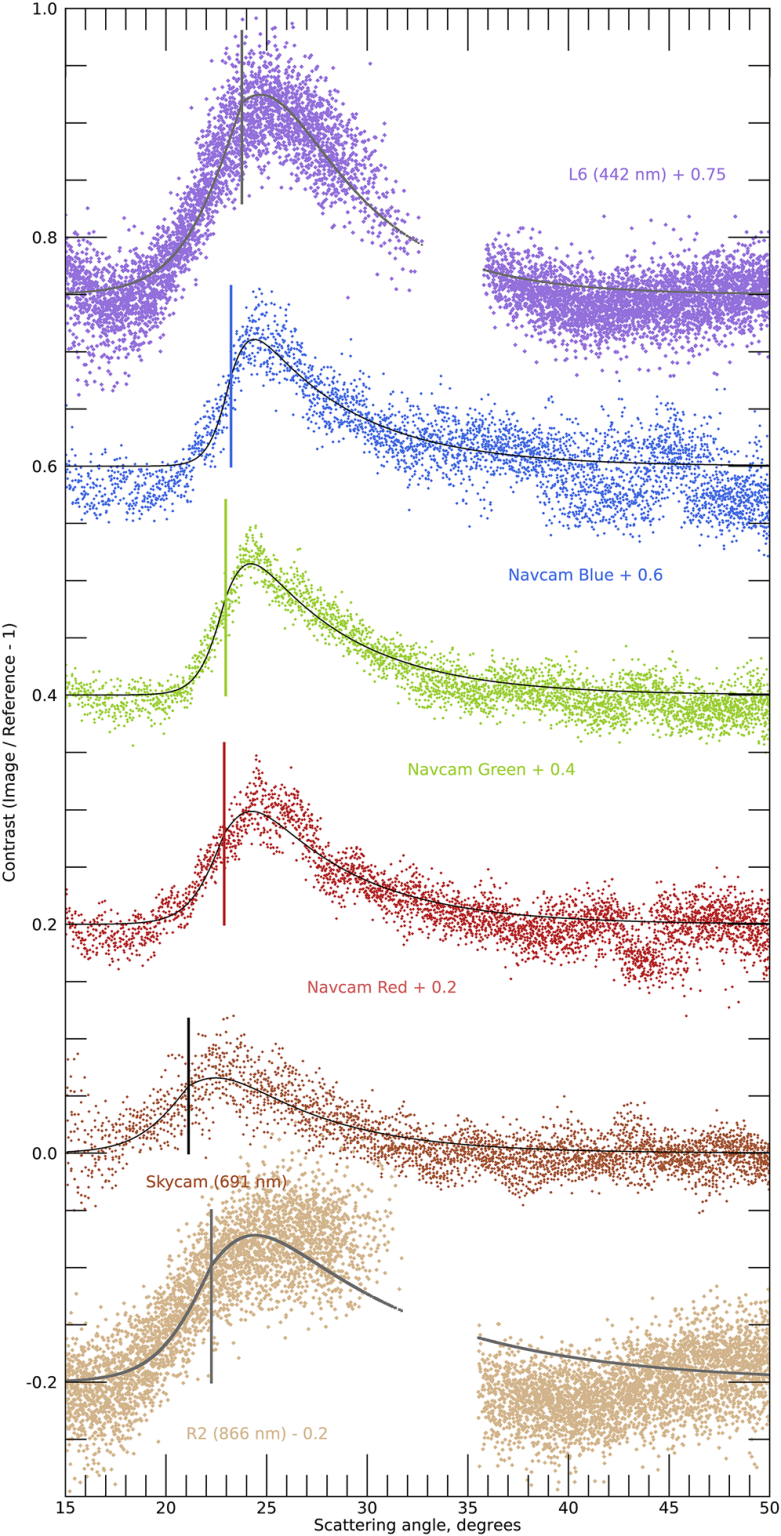
Halo brightness vs. scattering angle. From bottom: fractional residuals are shown for Mastcam‐Z R2 (tan), Skycam (brown), Navcam red, green, and blue channels, and Mastcam‐Z L6 (purple). Skycam and Navcam curves show sky above the Sun from sol 292 vs. 299 ratio images; Mastcam‐Z curves show a subsample of points from Figure 2. Each has a superposed model halo shape and vertical line indicating the fit inner edge of the halo.

The inner edge of the geometric‐optics halo varies with wavelength due to the changing index of refraction of water‐ice and is at 21.7° and 22.5° for red and blue light, consistent with our observations. Halos can be smoothed by both diffraction (for small particles) and roughness, such that the peak brightness can be outside 22°. We model the geometric‐optics halo as having a sharp inner edge and an exponential fall off to larger angles. We model broadening of the halo feature as being Gaussian in scattering angle. For the Navcam (Figure 1, right) and Skycam (Figure 2b) ratio images, we used a Levenberg‐Marquardt technique (Markwardt, 2008) to fit the model to the data with parameters for an inner edge, an amplitude, a fall‐off to large scattering angles, and a broadening. For the Mastcam‐Z images, we determined the ratio of the images to a simple model (an empirical fit to sky brightness that we have used for cloud searches, for which we excluded 20°–30° scattering angles from the fit, as shown in Figures 2c and 2d) and then determined the halo parameters as for the other cameras. Figure 4 shows the observed and model data for the 442 and 866 nm images. The best fit had an inner edge at 23.8° and 22.3°, with smoothing of 3°–5°, but the edge angles are likely uncertain by ∼1–2°. Contrast from Skycam (691 nm) and Navcam (630, 540, and 480 nm) ratio images (see Figures 1 and 2) are also shown in Figure 4. The best‐fit inner edges were at 21.1°, 22.9°, 23.0°, and 23.2°. Apparent smoothing declined from 1.3° to 0.6° with decreasing wavelength, roughly consistent with the diffraction of a sharp edge with a 40 to 60 μm scale. The later Mastcam‐Z images seemed to require larger smoothing, consistent with 8–9 μm diffraction scales, but which could instead relate to roughness or aggregation.

For terrestrial clouds at low temperatures (−75°C < *T* < −40°C), columns and rosettes form at high ice supersaturation (>20%), while lower supersaturation (1%–2% up to 10%–15%) results in irregular crystals and complex polyhedra (Bailey & Hallet, 2008). In Martian conditions, heterogeneous nucleation (growth on condensation nuclei such as the abundant dust) of water ice requires supersaturation above around 18% (Määttänen et al., 2005). Nucleation rate increases exponentially with increasing supersaturation up to around 100%. Thus, it is likely that the particles that formed the halo grew in a higher‐supersaturation environment than non‐halo‐forming clouds. A plausible mechanism is cooling from thermal tides, which could have a vertical extent of a scale height, and which may have interacted with gravity waves to produce a supercooled region of several km vertical scale. Alternatively, at a given rate, total ice volume increases proportionate to time spent in the supersaturated region, so a paucity of dust condensation nuclei coupled with a long period of growth might also produce sufficiently large particles. The relative efficiency of high supersaturation makes that our preferred hypothesis.

To form a halo of ∼10% contrast, we estimate that >1% of the optical depth must be halo‐forming ice, given factor‐of‐ten contrast in ideal halos (Yang et al., 2008). A much larger fraction, with a much lower‐contrast halo (i.e., due to diffraction or roughness) is permitted. For sol 292, the minimum large‐crystalline optical depth was 0.005, requiring as little as ∼0.03 Pr‐µm (precipitable‐microns) of water ice in the form of 11 μm columns. Total ice optical depth (estimated as optical depth in excess of background dust) was 0.1–0.3, requiring no more than 2–5 Pr‐µm of water ice for 11–30 μm columns in the absence of opacity from smaller particles. Ice optical depth was higher (0.4–0.6) on sol 296; this could also have been of order 1 Pr‐µm if it comprised only ∼3 μm particles.

The absence of prior halo detections may relate to the climatology of the ACB. No other landers have been as deep within the ACB: the larger, “Type 2” ice clouds of Clancy et al. (2003) occur from *L*_S_ 30°–140° at latitudes 0°–25°N. While there were two possible halos earlier, the sol‐292 halo detection was in an unusually cloudy phase at the end of ACB season. It is possible that halos could be seen once to several times annually at Perseverance's latitude. We expect that future cloud‐imaging campaigns by Perseverance will monitor more frequently for halos and that multi‐sensor follow‐up imaging will be rapidly employed following any future detection. We infer that halos are rare to nonexistent at the other landing sites based on the lack of reported observations and our inspection of thousands of ACB images with appropriate geometries. It remains unclear why halos were not seen during the Phoenix mission.

Ice sedimentation within halo‐forming clouds impacts the vertical distribution of both water and dust. We estimated settling velocities of between 0.3 and 1 m s^−1^ or timescales of a few hours—for 11 μm ice particles to fall one scale height near 40–50 km altitude. Particle diameters greater than 11 μm implies a dust vertical transport by cloud scavenging of about 10 km in a few hours, leading to dust clearing at the altitudes where the nucleation takes place.

## 4 Conclusions

We provide the first report of an optical (scattering) halo on another planet, in this case, a 22° halo caused by water ice. We show that on sol 292 of the Perseverance mission, a halo was observable over a 3.3 hr period of mid‐to late‐morning. The halo was detected by four cameras at six wavelengths. The halo, along with possible detections on the subsequent six sols, occurred during a cloudy and icy period that lasted from sols 285–300, during the ACB season. Two possible earlier halos were also associated with cloudy weather. The presence of a halo implied crystalline water‐ice particles grew to >11 μm in length and diameter as hexagonal prisms (columns). Associated RDS data suggested that the clouds started near 44 km altitude, although they could have precipitated by several km to more than a scale height during the morning. Halo‐forming clouds are likely rare due to the high supersaturation of water that is required but may be more common in northern subtropical regions during mid‐northern summer.

## Conflict of Interest

The authors declare no conflicts of interest relevant to this study.

## Supporting information

Supporting Information S1

## Data Availability

All Perseverance data used in this study are publicly available via the Planetary Data System (Bell & Maki, 2021; Maki, 2021; Rodriguez‐Manfredi & de la Torre Juarez, 2021). The subset of Mastcam‐Z products (Bell & Maki, 2021) used here include a sequence ID of ZCAM01xxx. The subset of Navcam (Maki, 2021) products used here include a sequence ID of NCAM00501. MEDA products (Rodriguez‐Manfredi & de la Torre Juarez, 2021) are organized by sol and subsystem. The sol range is defined in the text and all data files include a four digits sol in the filename.
