# Supplementary material for: Hexagonal Prisms Form in Water‐Ice Clouds on Mars, Producing Halo Displays Seen by Perseverance Rover
Source: Geophys Res Lett. 2022 Sep 9;49(17):e2022GL099776. doi: 10.1029/2022GL099776 (PMC9539710; doi:10.1029/2022GL099776)
Supplement: Supplementary file 1 — Supporting Information S1 [file GRL-49-e2022GL099776-s001.docx]

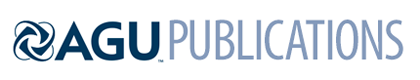


*Geophysical Research Letters*

Supporting Information for

Hexagonal Prisms Form in Water-ice Clouds on Mars, Producing Halo Displays Seen by Perseverance Rover

M.T. Lemmon^1^, D. Toledo^2^, V. Apestigue^2^, I. Arruego^2^, M.J. Wolff^1^, P. Patel^3^, S. Guzewich^4^, A. Colaprete^5^, Á. Vicente-Retortillo^2^, L. Tamppari^3^, F. Montmessin^6^, M. de la Torre Juarez^3^, J. Maki^3^, T. McConnochie^1^, A. Brown^7^, J.F. Bell III^8^

^1^Space Science Institute, Boulder, CO 80301, USA. ^2^Instituto Nacional de Técnica Aerospacial, Madrid, Spain. ^3^Jet Propulsion Laboratory, California Institute of Technology, Pasadena, CA, USA. ^4^Goddard Space Flight Center; Greenbelt, MD, USA. ^5^NASA Ames Research Center, Moffett Field, 94035, CA, USA. ^6^LATMOS, Paris, France. ^7^Plancius Research, MD, USA. ^8^Arizona State University, Tempe, AZ, USA.

**Contents of this file**

Text S1. Supporting images.

Figure S1. Navcam images over sols 292-308.

Figure S2. Skycam images over sols 289-300.

Text S2. RDS detection.

Figure S3. Representation of the LAT4 view for the halo detection for sol 292.

Figure S4. RDS LAT4 signal from sols 292 and 295 with both same rover attitude.

Text S3. Cloud images.

Figure S5. Clouds imaged by Navcam.

Text S4. RDS radiative-transfer analysis and particle sedimentation.

Figure S6. RDS observations at dawn on sol 292.

Figure S7. Sedimentation of ice crystals.

**Introduction**

Supporting information includes comparisons of additional sols of Navcam and Skycam imaging (S1); a description of the RDS detection of the halo on the morning of sol 292 (S2); an example of the cloudy images that were taken during sols near 292 (S3); and a description of the RDS radiative-transfer analysis used for altitude constraint.

Text S1. Supporting images.

The sol 292 Navcam images were compared to the full set of follow-up images to search for additional halos, with no detection (Fig. S1). Sol 289-300 Skycam images were compared, with possible weak halos over sols 293-298 (Fig. S2).


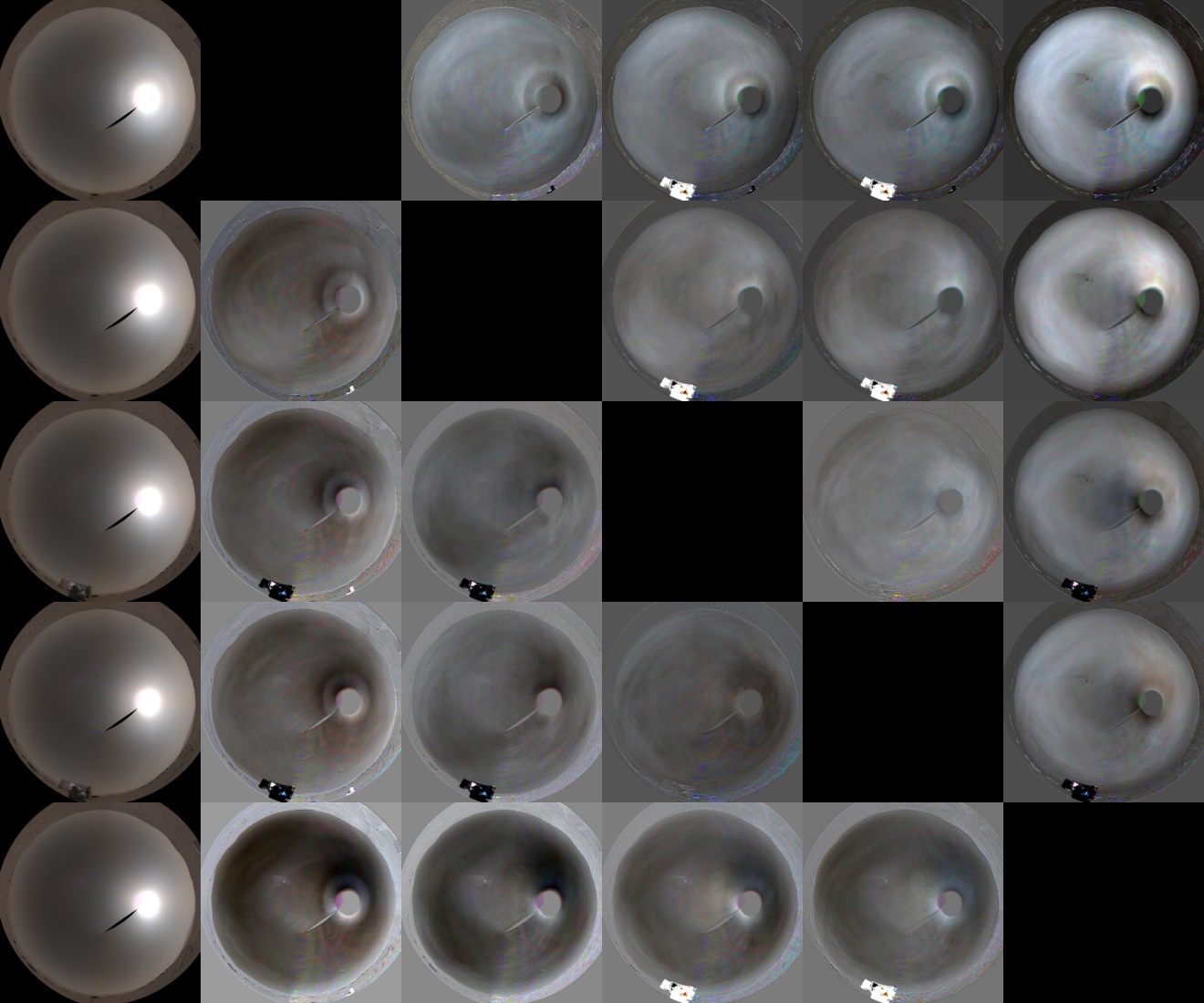
Figure S1. Navcam images over sols 292-308. The left column shows equidistant projections for sols 292, 299, 303, 304, and 308 (from top). The next column to the right is the ratio of the sol 292 image to each image from column 1. Each successive column is the next image ratioed to the others.


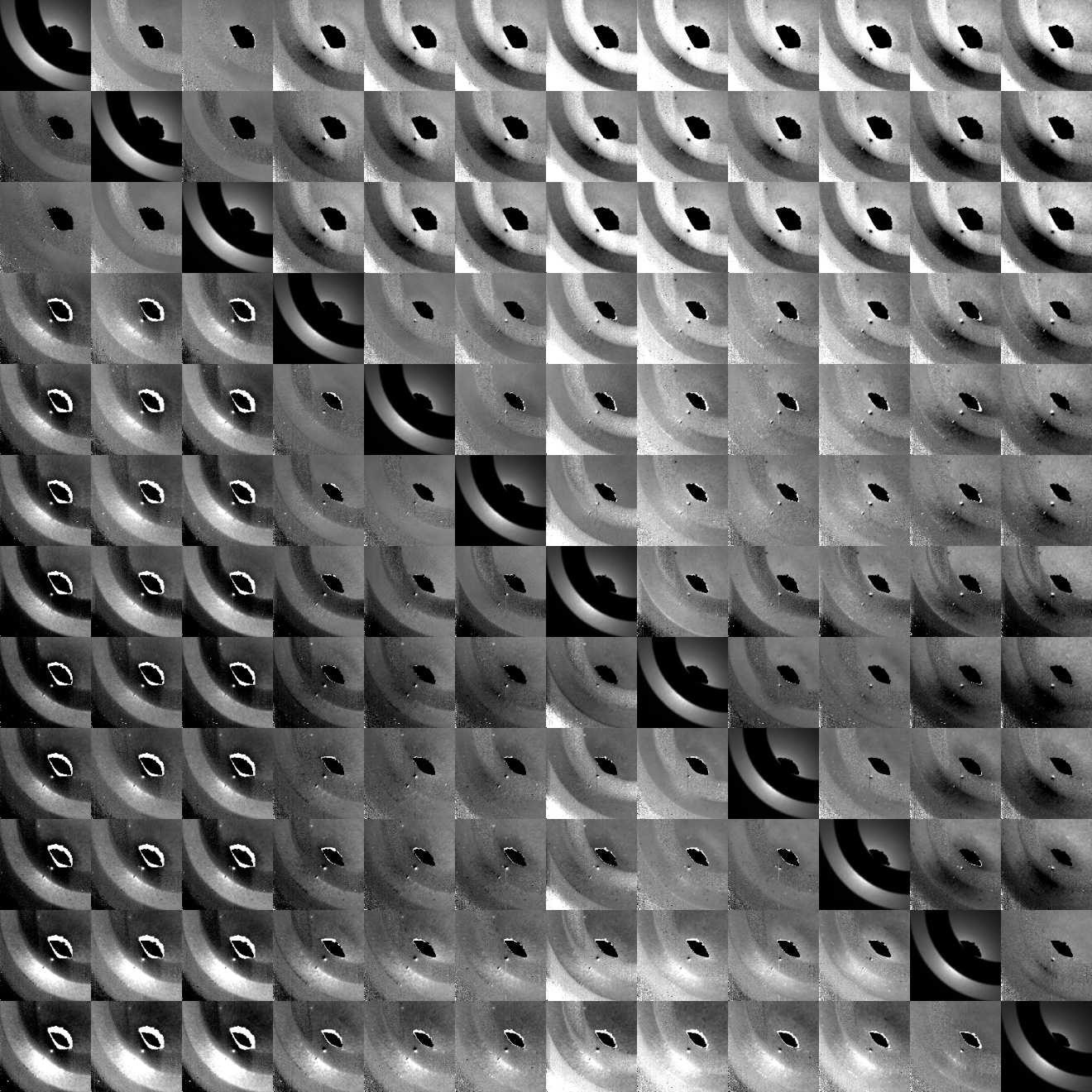
Figure S2. Skycam images over sols 289-300. Along the diagonal, successive images (East-facing quadrant) are (from upper left) from the mornings of sol 289, 290, 291, 292, 293, 294, 295, 296, 297, 298, 299, and 300. The remaining frames show ratios of the images (left to right in the numerator, top to bottom in the denominator).

Text S2. RDS detection.

The MEDA RDS includes two sets of 8 photodiodes. One set is pointed upward (referred as top sensors), with each photodiode covering a different wavelength range between 250-1000 nm. The other set is pointed sideways (lateral sensors), 20° above the horizon, and they are spaced 45° degrees apart in azimuth to sample all directions at a single wavelength. For sol 292, RDS signal corresponding to lateral 4 showed an increase in brightness at around 08:23 LTST, a time when the sun is about 25° away from this sensor maximum-of-transmission. Figure S3 shows the interference view of the lateral-4 sensor (black represents 0% of transmission) along with the sensor FOV (represented as a color disk). The white dot indicates the sun position relative to RDS at 08:11 LTST, and the white ring around the sun represents a halo with a radius of 22°. Figure S4 shows the variation of lateral-4 signal with the angular distance between the sun and the sensor measured on sol 292. For comparison purposes the signal for sol 295 (with no presence of halo and same rover attitude) is also represented. With respect to sol 295, we saw an increase in the signal of about 2.87 % at 25°. It is important to note here that the sensor FOV is about ±4.5° (FWHM), and thus this explains why the maximum is extended about ~ 5° around the 25°. On the other hand, the net effect of the halo over RDS signal depends on both the angular distance between the halo and the sensor maximum-of-transmission, and the contribution of direct and scattered light at the other angles (that is to say, the level of light without the halo). These two effects explain why the maximum in RDS signal occurs at 25° and not at the angle when the halo is crossing the sensor maximum-of-transmission (22°). Indeed, if we take sol 295 as reference the decrease in the background signal is about 20 % when the angular distance between the halo and the sensor maximum-of-transmission varies from 22 to 25°.


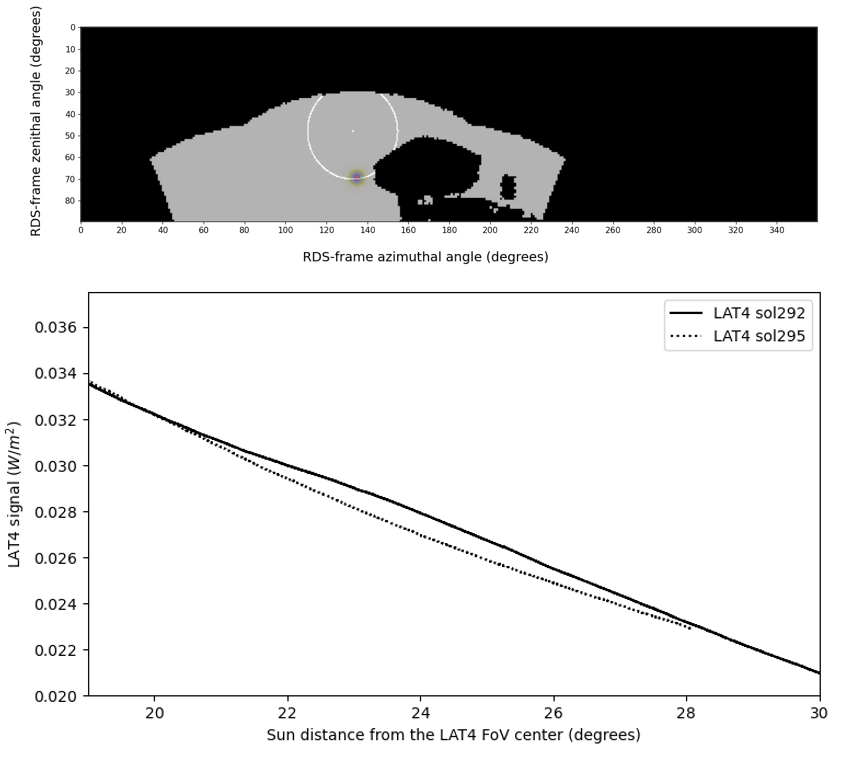
Figure S3. Representation of the LAT4 view for the halo detection for sol 292.


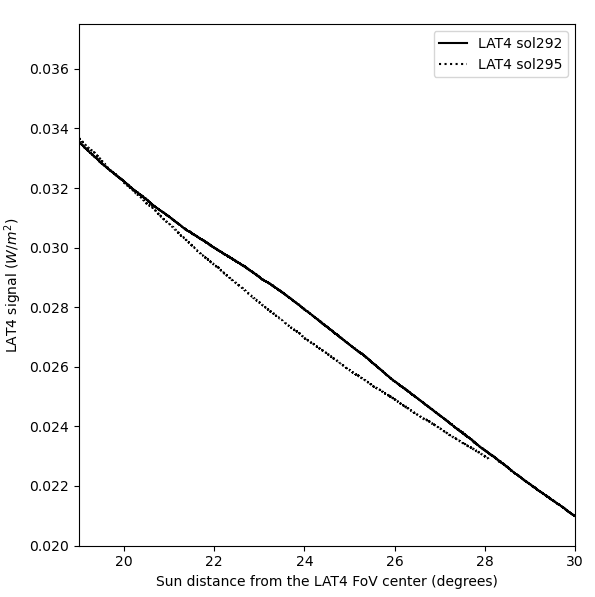


Figure S4. RDS LAT4 signal from sols 292 and 295 with both same rover attitude.

Text S3. Cloud images.

The NavCam instrument has also been used to collect cloud movies that are processed using a Mean Frame Subtraction (MFS) method to enhance cloud features. This involves taking an average frame of the entire movie which is then subtracted from individual frames from the movies. This enhances the time-variable component in the movie revealing cloud features and cloud movement within the frames. Using this, we observed a large amount of water ice clouds over Jezero crater with peak activity seen between sol 282 to sol 304, around the time the halo was observed.


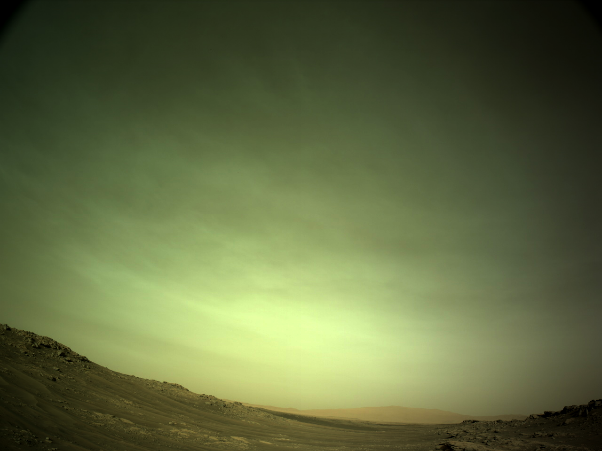

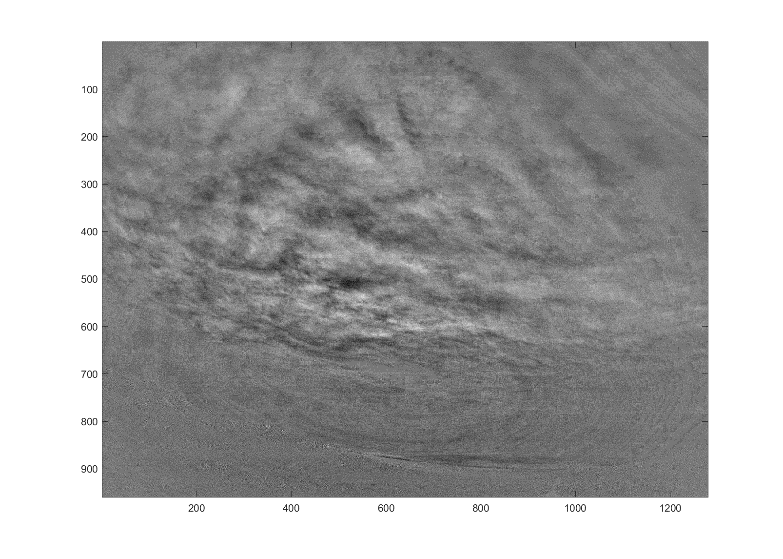


Figure S5. Clouds imaged by Navcam. This shows an example of raw versus MFS processed frames of a NavCam cloud movie. These images are specifically from sol 292. The cloud movies taken on sol 292 show clouds over Jezero that appear thicker and brighter than on the clouds seen earlier in the season.

Text S4. RDS radiative-transfer analysis and particle sedimentation.

A Monte-Carlo radiative transfer code in spherical geometry (required for SZA>90°) was used to simulate the scattering of Mars atmosphere at twilight. RDS signals at zenith at 450 and 950 nm measured during the sunrise of sol 292 were fitted simultaneously using a two-layer aerosol model compromising a dust layer vertically extended following the Conrath profile, and a cloud layer whose altitude and particle radius are free parameters. To decrease the dependence of the simulations with dust properties, the signals were normalized to their respective values at 90° (in this case the signal variation with SZA mainly depends on the aerosol vertical structure). This analysis provides the best fit shown in Fig S6 with cloud altitude=44.32 km and cloud particle radius=4.71 μm. If we define the errors of these parameters through the ${\Delta\chi}^{2}$ corresponding to the 3σ significance level, then we obtain cloud altitudes between 43 and 50 km and cloud particle sizes between 3 and 30 μm. Although the cloud altitude is better constrained than the particle size, this analysis indicates that a cloud layer made of submicron particles cannot reproduce RDS observations, and thus the halo should be produced by water ice particles.

The settling velocities for cloud and dust particles were computed by:

$v=\frac{2g\rho_{p}}{9\upsilon_{a}}\left\{ 1+Kn\left[ 1.246+0.42e^{\frac{-0.87}{Kn}} \right] \right\}r^{2}$

Where g is gravity, $\rho_{p}$ is the particle density, ν_a_ is the air dynamic viscosity, Kn is the Knudsen number and r the particle radius. Note that the term between the brackets represents the Cunning-ham slip-flow correction. Figure S7 show the variation of the settling velocities and sedimentation timescales with height for cloud particles for different particle sizes. For these velocities we used a particle density of 917 km m^-3^ for water ice particles and of 2500 km m^-3^ for dust. As the settling velocities depend on temperature and this parameter is highly variable, the same velocities were estimated for $\Delta T=\pm5^{\circ}$, whose variations are expressed by the horizontal errorbars. We also made computations for cylinder-type particles with different aspect ratios (and for Reynolds numbers not too small) and found similar results for mass-equivalent spheres.

Figure S6. RDS observations at dawn on sol 292. Comparisons between radiative-transfer model and normalized RDS observations at sunrise on sol 292. The blue lines with errors represent RDS observations at zenith at 450 nm (left panel) and 950 nm (right panel). For comparison purposes, RDS signals measured for a cloud-free day are also shown (black solid line). The two signals were fitted simultaneously using the dust + cloud model discussed above (red solid line).

Figure S7. Sedimentation of ice crystals. Variation of settling velocity (left panel) and sedimentation timescale (right panel) with altitude for cloud particles with radii of 9.78, 19.57 and 58.78 μm. These spherical particles are equivalent in mass to cylinders with r=5, 10 and 30 μm, and 2r/L=0.1. The errors were computed assuming ΔT=5 K, and the water ice density was set to 917 kg m^-3^. Similar computations were made for r=5, 10 and 30 μm, and 2r/L=0.1, and we found velocities by about 1.7 smaller than those for 2r/L=0.1.
